# Do malignant self-regard and depressive personality account for appearance evaluation? Preliminary results

**DOI:** 10.1192/j.eurpsy.2022.1430

**Published:** 2022-09-01

**Authors:** R. Cavalli, G. Rogier, P. Velotti

**Affiliations:** 1 University of Rome Sapienza, Dynamic And Clinical Psychology And Health, Rome, Italy; 2 University of Genoa, Department Of Educational Sciences, Genoa, Italy

**Keywords:** depressive personality, appearance evaluation, malignant self-regard

## Abstract

**Introduction:**

Despite the growing number of studies focusing on the relationship between appearance evaluation and personality dimension, few is known regarding the role of depressive personality and malignant self-regard regarding this topic. Moreover, there is a lack of studies investigating the potential role of both clarity of self-concept and interpersonal exclusion feelings in this relationship.

**Objectives:**

To extend the knowledge regarding the relationships between malignant self-regard, depressive personality and appearance evaluation.

**Methods:**

We administered to a very large sample of adults a battery of self-report questionnaires including the subscale Appearance evaluation of the Multidimensional Body-Self Relations Questionnaire, the Self Concept Clarity Scale, the Malignant self-regard questionnaire, the Depressive Personality Inventory and the Core Exclusion Schema Questionnaire.

**Results:**

We found that depressive personality negatively predicted positive appearance evaluation whereas the inverse pattern of results was obtained in relation to malignant self-regard. Moreover, we found that both poor self-concept clarity and feelings of exclusion mediate the relationship between malignant self-regard and positive appearance evaluation.

**Conclusions:**

Depressive personality and Malignant self-regard appear to be promising construct to investigate in the field of eating disorders.

**Disclosure:**

No significant relationships.

